# Chromatographic Fingerprint Analysis of Marrubiin in *Marrubium vulgare* L. via HPTLC Technique

**DOI:** 10.15171/apb.2016.019

**Published:** 2016-03-17

**Authors:** Keyvan Yousefi, Sanaz Hamedeyazdan, Mohammadali Torbati, Fatemeh Fathiazad

**Affiliations:** ^1^ Department of Pharmacology, Faculty of pharmacy, Tabriz University of Medical Sciences, Tabriz, Iran.; ^2^ Department of Pharmacognosy, Faculty of pharmacy, Tabriz University of Medical Sciences, Tabriz, Iran.; ^3^ Department of Traditional Pharmacy, Faculty of Traditional Medicine, Tabriz University of Medical Sciences, Tabriz, Iran.

**Keywords:** Marrubium vulgare, Marrubiin, Folin-Ciocalteau, Free radicals, TLC scanner, Densitometry

## Abstract

***Purpose:*** In the present study we aimed to quantify marrubiin, as the major active compound, in the aerial parts of *Marrubium vulgare* from Iran using a HPTLC-densitometry technique.

***Methods:*** Quantitative determination of marrubiin in *M. vulgare* methanol extract was performed by HPTLC analysis via a fully automated TLC scanner. Later on, the in vitro antioxidant activity of the *M. vulgare* methanol extract was determined using 1,1-diphenyl-2-picryl-hydrazil (DPPH) free radical scavenging assay. Furthermore, total phenolics and flavonoids contents of the methanol extract were quantified, spectrophotometrically.

***Results:*** The amount of marrubiin was calculated as 156 mg/g of *M. vulgare* extract. The antioxidant assay revealed a strong radical scavenging activity for the *M. vulgare* methanol extract with RC_50_ value of 8.24μg/mL. Total phenolics and flavonoids contents for *M. vulgare* were determined as 60.4 mg gallic acid equivalent and 12.05 mg quercetin equivalent per each gram of the extract, correspondingly.

***Conclusion:*** The presented fingerprint of marrubiin in *M. vulgare* extract developed by HPTLC densitometry afforded a detailed chemical profile, which might be useful in the identification as well as quality evaluation of herbal medications based on *M. vulgare*. Besides, the considerable antioxidant activity of *M. vulgare* was associated with the presence of marrubiin along with phenolics and flavonoids exerting a synergistic effect.

## Introduction

*Marrubium vulgare* (Lamiaceae), known as horehound in English and Khanak in Persian, is a popular medicinal herb which has been used traditionally as a remedy for a wide range of diseases such as hypertension, infections, pain and etc.^1,2^* M. vulgare* shows strong antioxidant activity due to the presence of flavonoids, terpenes, and phenols.^3^ The main active ingredient of *M. vulgare* was found to be marrubiin which has been identified in 1984 as the first diterpene isolated from leaves of this plant. Marrubiin is a furan labdane diterpenoid which is produced and accumulated only in the aerial parts of the plant.^1,4^ It is important to mention that marrubiin is generated as an artifact from pre-marrubiin during the extraction procedure when heat is involved in the extraction or concentration procedure.^5^ Marrubiin has been associated with the bitter principle of the horehound and lots of other medicinal plants of the family Lamiaceae. The broadly known diterpenoid lactone, marrubiin, has been marked with assorted types of biological activities such as analgesic, vasorelaxant, cardioprotective, gastroprotective, antidiabetic, antioxidant, antispasmodic, anti-hypertensive, anti-edematogenic and immunomodulating properties.^1,6-15^ In a study conducted by Mnonopi *et al,* marrubiin which was isolated from *Leonotis leonurus* L. found to be a cardioprotective compound by inducing anticoagulant, antiplatelet and anti-inflammatory properties in obese rat models.^6^ Our previously published paper on cardioprotective effect of the total methanolic extract of *M. vulgare,* containing marrubiin as the major ingredient of the extract, in an animal model with myocardial infarction was in consistence with the reported activity related to the compound marrubiin.^16^ Furthermore, in a recent study gastroprotective activity of the methanolic extract of *M. vulgare* had been revealed due to the presence of marrubiin as evidenced by inhibitory effect of both marrubiin and the methanol extract of *M. vulgare* on the indomethacin-induced ulcers.^17^ Generally, considering loads of published data over the past century on the chemical and biological aspects of marrubiin in the genus *Marrubium* (Lamiaceae), we could consider this compound as a biomarker within the plants of this genus.

In view of these facts, High Performance Thin Layer Chromatography (HPTLC) has been widely used as a rapid, precise and cost-effective method for determination of biological compounds from medicinal plants; accordingly, in this study we adopted HPTLC as a promising technique for determination of marrubiin quantitatively within the *M. vulgare* extract.^18^ It is of note to mention that HPTLC encompasses the use of chromatographic layers of utmost separation efficiency, utilization of instrumentation for all steps in the approach, defined sample applications, validated reproducible chromatogram developments and software controlled analysis. Nonetheless, adaptation on optimized new approaches would not be inevitable in displacement of existing methods but a supplement to already existing techniques. The standardized method might be useful in identification as well as quality evaluation of herbal medications based on a specific phytochemical. Following our previous work, in the present study a preliminary phytochemical screening of the methanol extract of *M. vulgare* from Iran was performed. Additionally, an HPTLC-densitometry assay was conducted for the purpose of quantitative determination of marrubiin content in the extract.

## Materials and Methods

### Materials

All the chemicals, including solvents, were of analytical grade from Merck Company Germany. 1,1-Diphenyl-2-picryl-hydrazyl (DPPH), quercetin, gallic acid, Folin-Ciocalteu reagent, and aluminum chloride from Sigma-Aldrich chemical company Madrid-Spain, potassium acetate, and silica gel 60 F_254_ HPTLC (20cm × 20cm) plates (Merck, Darmstadt, Germany) were used in this study.

### Plant Material, Extraction and Preparation

The aerial parts of *M. vulgare* were collected in 2012 during flowering stage on June from Kiasar (Mazandaran Province, Iran). They were authenticated by Dr. M. Mazandarani (Department of Biology, Azad Islamic University, Gorgan, Iran). Voucher specimens (No. 712 Tbz-Fph) have been deposited at the herbarium of the Department of Pharmacognosy, Faculty of Pharmacy, Tabriz University of Medical Sciences, Tabriz, Iran. Air dried aerial parts (200g) were grounded and extracted with methanol (2L×4) by maceration at room temperature (25-30 °C). The obtained extract was concentrated to dryness under vacuum at 40 °C using a rotary evaporator. A greenish residue weighing 17.8 % (w/w) was obtained and kept in air tight bottle in a refrigerator until use. To identify the chemical constituents, the resultant methanol extract was subjected to preliminary phytochemical analysis.

### Determination of Total Phenolic Content

Total phenolic content was determined by Folin-Ciocalteau method as described by Ghasemi *et al* and Ebrahimzadeh *et al*.^19,20^ Briefly, 0.5 ml of the extract was mixed with Folin-Ciocalteau reagent (5 mL, 1:10 diluted with distilled water) for 5 min and aqueous Na_2_CO_3_ (4 mL, 1 M) was then added. The mixture was allowed to stand for 15 min where the phenolics were determined by colorimetry at 765 nm (Shimadzu 2100, Japan). The standard curve was prepared by 50, 100, 150, 200, and 250 mg/mL solutions of gallic acid in methanol: water (50:50, v/v). Total phenol values are expressed in terms of gallic acid equivalent (mg/g of the extract) by reference to calibration curve: y=0.0067x+0.0132, (R^2^=0.987).

### Determination of Total Flavonoids Content

In order to determine the total flavonoids content of the *M. vulgare* extract, the colorimetric aluminum chloride method was employed according to the method described by Ghasemi *et al* and Ebrahimzadeh *et al*.^19,20^ Briefly, 0.5 mL solution of methanolic extract were mixed with 1.5 mL of methanol, 0.1 mL of 10% aluminum chloride, 0.1 mL of 1 M potassium acetate and 2.8 mL of distilled water, and were left at room temperature for 30 min. The absorbance of the reaction mixture was measured spectrophotometrically at 415 nm. Total flavonoids content were calculated as quercetin from a calibration curve prepared using 31.25-250 μg/mL solutions of quercetin in methanol as standard (y=0.008x-0.068, R^2^=0.999).

### Determination of In Vitro Antioxidant Activity

The free radical scavenging capacity of the extract was measured from the bleaching of the purple colored methanol solution of 1,1-diphenyl-2-picryl-hydrazil. The stock concentration 1 mg/ml of the methanol extract of the *M. vulgare* was prepared followed by dilution in order to obtain concentrations of 5×10^-1^, 2.5×10^-1^, 1.25×10^-1^, 6.25×10^-2^, 3.13×10^-2^ and 1.56×10^-2^ mg/mL. The obtained concentrations in equal volumes of 2 mL were added to 2 mL of a 0.004% of DPPH solution. After a 30 min of incubation period at 25 ºC, the absorbance at 517 nm was determined against a blank. Tests were carried out in triplicate where the average absorption was noted for each concentration. Furthermore, as the positive control the same procedure was repeated with quercetin. The inhibition percentages of DPPH free radicals of by the samples were calculated following the equation:

R (%) = 100 × [(A blank – A sample) / A blank]

Hereon, “A blank” represents the absorbance value of the control reaction and “A sample” is the absorbance value for each sample. Besides, RC_50_ value, concentration of the extract reducing 50% of the DPPH free radicals, was calculated from the graph of inhibition percentages against concentrations of *M. vulgare* extract in mg/mL.

### Quantitative Analysis of Marrubiin

Preliminary HPTLC analysis of the *M. vulgare* extract was performed on silica gel 60 F_254_ HPTLC plate with benzene-acetone (17:3) as the mobile phase. Later on, detection of the spots after spraying with anisaldehyde-sulfuric acid reagent revealed the presence of marrubiin at RF=0.82 as the major compound in the extract such as expressed by Popa et al, in 1968. Following the preliminary qualitative analysis of HPTLC analysis of the *M. vulgare* extract, quantitative determination of marrubiin, as the major bioactive component in *M. vulgare,* was performed by photodensitometric method via a fully automated TLC scanner (CAMAG TLC scanner 3 coupled with Automatic TLC Sampler 4, Automatic Developing chamber 2 and a TLC Visualizer). In addition, winCATS (Planar chromatography manager) software was used for analyzing results of the plate scan.

As far as we know, automatic sample application is a key factor for productivity of the HPTLC laboratory evaluations, to this end; Automatic TLC Sampler 4 was used in spraying samples onto plates in the form of bands in the presence of nitrogen air. In this regard, samples were applied on the plate as 4 mm wide bands with constant application rate from 1 to 7µL s^-1^, an automatic TLC sampler under a flow of N_2_ gas, 20 mm from the bottom, 15 mm from the side, and spaces among the spots were 8 mm of the plate. In order to quantitative determine the content of marrubiin in the extract and obtain a standard curve, stock solution of marrubiin was prepared in methanol to achieve the concentration of 0.44 mg/ml. Samples of marrubiin on the HPTLC plate with volume of 1µl to 7µl were consequently spotted to afford 0.44, 0.88, 1.31, 1.75, 2.19, 2.63 and 3.06 µg marrubiin (spots number 1 to 7). Similarly, a stock solution of extract in methanol (10 mg/ml) was prepared and spotted with three volumes of 1, 2 and 3µl immediately after standard solutions' spots (spots number 8 to 10). Subsequently, the linear ascending development was carried out in a twin trough chamber, which was pre-saturated with 25 mL mobile phase with benzene-acetone (17:3) for 30 min, at room temperature (25 ± 2 °C) and 50 ± 5% relative humidity. The developing chamber of the HPTLC system automatically performed the development step minimizing environmental effects. Thus, the activity and preconditioning of the layer, chamber saturation, developing distance and final drying were completely pre-set and monitored during this step. When the Linomat is operated under software, plate dimensions, number and distance of tracks, designation, sample volumes and sequence are software controlled. All the operating data were automatically transferred to the densitometric processing evaluation step. Ultimately, the HPTLC plate was placed on a TLC Scanner and scanned under the UV 509 nm light (A 509-nm UV light was used to illuminate the plate).

## Results

### Phytochemical Screening of M. vulgare Extract

Preliminary phytochemical screening of *M. vulgare* extract indicated the presence of flavonoids and phenolic compounds in the plant extract. Regarding the absorbance values of the extract solutions, reacted with Folin-Ciocalteu and aluminum chloride reagents compared to the standard solutions as described in the methods section, the amount of total phenolic and flavonoids contents for *M. vulgare* were determined as 60.4 mg gallic acid equivalent/g extract and 12.05 mg quercetin equivalent/g of the extract, respectively.

### In Vitro Antioxidant Activity of M. vulgare Extract

The free radical scavenging activity of *M. vulgare* extract was evaluated using the DPPH method in vitro. According to our results, the RC_50_ values for methanol extract of *M. vulgare* and quercetin were found to be 8.24 and 3μg/ml, correspondingly. Our results showed that the *M. vulgare* methanol extract has a considerable free radical scavenging activity comparable with the standard compound, quercetin.

### Marrubiin Quantification

**Figure 1:**
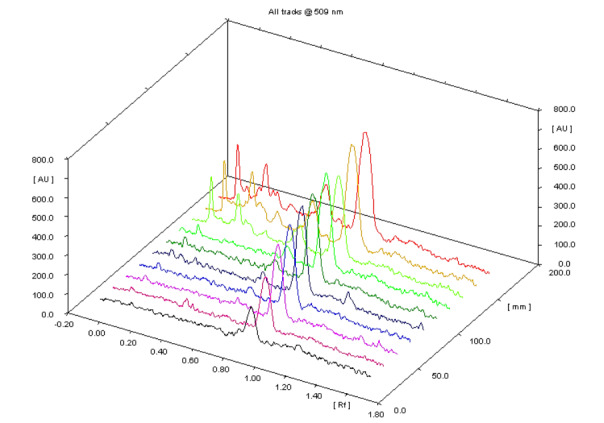
Photodensitogram of the fluorescent HPTLC plate of marrubiin (densitograms 1-7) and *M. vulgare* extract (densitograms from 8-10) depicted by TLC scanner 3 (CAMAG) in which the X, Y and Z axis represents RF of each detected spot, height of the peaks (Spot’s density), and location on the plate, respectively.

**Figure 2:**
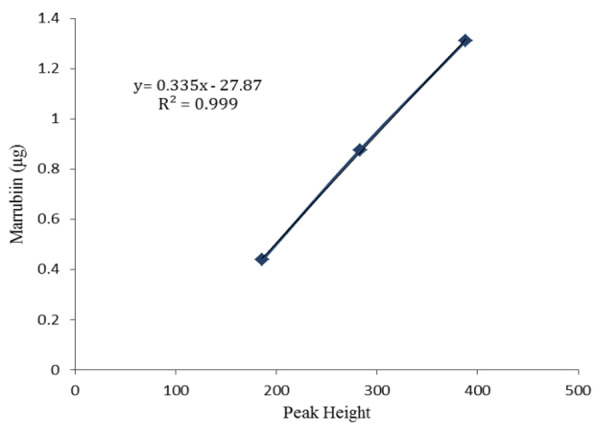
The calibration curve drawn automatically by TLC Scanner used to quantify marrubiin in the methanol extract of *M. vulgare*.

Preliminary HPTLC analysis of the extract revealed marrubiin with R_f_ value of 0.82 as a major compound present in the *M. vulgare* extract. The quantitative determination of marrubiin on the fluorescent HPTLC plate on TLC scanner yielded a 3D graph on the basis of optical density (densitogram). In Figure 1, densitograms 1 to 7 are the increasing concentrations of pure compound marrubiin. The calibration curve was calculated automatically according to the densitograms 1 to 7 that in this curve the Y axis is marrubiin concentration and the X axis is the peak height of the corresponding concentration (Figure 2). The densitograms 8 to 10 are three repeated samples of the methanol extract from aerial parts of *M. vulgare* where the mean value of the heights of these three points were used to quantify the marrubiin content in the extract. Eventually, the amount of Marrubiin was calculated as 156 mg/g of *M. vulgare* extract by reference to the standard curve: (y=0.335x-27.87, R^2^= 0.999) (Figure 2).

## Discussion

Our preliminary phytochemical findings in this study revealed the presence of phenolic compounds, flavonoids and marrubiin as the major diterpenoid in the methanolic extract of *M. vulgare*. It was found that the considerable radical scavenging activity of the extract against the DPPH free radicals was in line with the high phenolic and flavonoid contents of *M. vulgare* extract. The naturally occurring flavonoids and phenolics are believed to possess the ideal chemical structure for scavenging free radicals.^21,22^

Owing to the fact that free radicals are molecules with an odd, unpaired electron that these unpaired electrons make them unstable and highly reactive,^23^ they could be extremely toxic to human cells attacking fatty acids, leading to lipid peroxidation of membranes, reacting with proteins, destruction and oxidation of amino acids, oxidation of sulfhydryl groups and polypeptide chain scission. Accordingly, these free radicals are the important factors in diseases related to oxidative stress conditions such as cardiovascular and neurodegenerative diseases.^24^ The use of natural antioxidants, especially phenolics and flavonoids, might be very promising in treatment of these kinds of diseases. Hence, in recent years more investigations have been focused on the plants especially those with remarkable antioxidant activities.^22,25^ Overall, here in this study it was suggested that the observed scavenging activity of the *M. vulgare* methanol extract could be assigned to the hydrogen-donating capacity of the phenolic and flavonoid components, in cooperation with the presence of marrubiin in the extract.

Formerly in a report by our team, the methanol extract of *M. vulgare* showed significant improvements to the hemodynamic, electrocardiographical and histopathological changes in a myocardial infarction model induced by isoproterenol.^16^ However, our new findings in this paper provided sufficient evidence for the presence of high quantities of a diterpenoid marrubiin along with flavonoids and phenolics corroborating the cardioprotective effect of *M. vulgare.* Furthermore, we can come to the conclusion that these compounds might have a synergistic antioxidant effect through which they induce protection against oxidative stress. Taken together, *M. vulgare* from Iran have the potential to be considered as a suitable applicant to be probed as a source of bioactive components in search of new efficient versatile phytomedicines for treating various ailments especially those caused by oxidative stress.

On the other hand, a high performance thin layer chromatography method coupled with densitometric analysis was developed in this study for the identification and quantification of marrubiin in *M. vulgare.* This method was considered to be precise, consistent, rapid and low-cost which can be used for quantitative determination of marrubin in the extracts from *Marrubium* species. According to the findings, the presented chemical fingerprint of *M. vulgare* extract that was developed using HPTLC densitometry afforded a detailed chemical profile which might be useful in the identification as well as quality evaluation of herbal medications based on *M. vulgare*. As follows, the developed fingerprint could be of value in preparation of a standardized herbal product with consistent biological activity. Likewise, providing these kinds of chemical fingerprints may also be helpful in differentiation of the plant from adulterants according to the simplicity, flexibility and cost efficiency of the HPTLC technique in both qualitative and quantitative aspect with minimal time requirement.

## Conclusion

Regarding the phytochemical findings of the present study it can be concluded that the sizeable radical scavenging activity of *M. vulgare* methanol extract could be attributed, to some extent, to its remarkable flavonoids and phenolics content. Moreover, it was revealed that marrubiin was the main compound in the aerial parts of *M. vulgare* grown in Iran. The quantification of marrubiin could be performed using HPTLC technique which is a precise, simple and relatively inexpensive method. According to our knowledge, there is no report on this method for analyzing marrubiin in the genus *Marrubium* so far. Thereby, this method could be used simply and rapidly for the routine analysis and quality control of marrubiin in the *Marrubium* species.

## Acknowledgments

The authors would like to thank Biotechnology Research Center of Tabriz University of Medical Sciences for their help in conducting the HPTLC analysis. Financial support of this work by the Research Vice-Chancellor of Tabriz University of Medical Sciences is faithfully acknowledged. Authors are also thankful to Dr. M. Mazandarani for authenticating the plant.

## Ethical Issues

Not applicable.

## Conflict of Interest

The authors report no conflicts of interest.
